# Diffuse axonal injury in brain trauma: insights from alterations in neurofilaments

**DOI:** 10.3389/fncel.2014.00429

**Published:** 2014-12-17

**Authors:** Declan G. Siedler, Meng Inn Chuah, Matthew T. K. Kirkcaldie, James C. Vickers, Anna E. King

**Affiliations:** ^1^Wicking Dementia Research and Education Centre, Medical Sciences PrecinctHobart, TAS, Australia; ^2^School of Medicine, University of TasmaniaHobart, TAS, Australia

**Keywords:** traumatic brain injury, diffuse axonal injury, neurofilament, NFL, diffuse brain trauma, traumatic axonal injury, biomarkers, neurofilament compaction

## Abstract

Traumatic brain injury (TBI) from penetrating or closed forces to the cranium can result in a range of forms of neural damage, which culminate in mortality or impart mild to significant neurological disability. In this regard, diffuse axonal injury (DAI) is a major neuronal pathophenotype of TBI and is associated with a complex set of cytoskeletal changes. The neurofilament triplet proteins are key structural cytoskeletal elements, which may also be important contributors to the tensile strength of axons. This has significant implications with respect to how axons may respond to TBI. It is not known, however, whether neurofilament compaction and the cytoskeletal changes that evolve following axonal injury represent a component of a protective mechanism following damage, or whether they serve to augment degeneration and progression to secondary axotomy. Here we review the structure and role of neurofilament proteins in normal neuronal function. We also discuss the processes that characterize DAI and the resultant alterations in neurofilaments, highlighting potential clues to a possible protective or degenerative influence of specific neurofilament alterations within injured neurons. The potential utility of neurofilament assays as biomarkers for axonal injury is also discussed. Insights into the complex alterations in neurofilaments will contribute to future efforts in developing therapeutic strategies to prevent, ameliorate or reverse neuronal degeneration in the central nervous system (CNS) following traumatic injury.

## Introduction

Diffuse axonal injury (DAI), regarded as an integral process in all grades of traumatic brain injury (TBI), results typically from head rotational acceleration/deceleration, as well as the propagation of force through the brain following impact (Adams et al., 1989; Browne et al., 2011; Gupta and Przekwas, 2013). It is characterized by two distinct types of axonal pathology: swellings or varicosities along the length of axons, and the presence of large terminal bulbs (Chen et al., 1999; Smith et al., 2003; Johnson et al., 2013). Progressive, post-traumatic swelling of these structures leads to axonal disconnection (Povlishock and Christman, 1995). These swellings contain neurofilament accumulations (Okonkwo et al., 1998; Huh et al., 2002; Marmarou and Povlishock, 2006; DiLeonardi et al., 2009), but the role neurofilaments play in the development and progression of damage is yet to be elucidated. Uniaxial tension is associated with the development of varicosities in the long white matter tracts, whereas sudden shear forces have been linked with the development of axonal bulbs at gray-white matter interfaces, with relative preservation of the proximal tracts (Chen et al., 1999; Smith et al., 2000). Diffuse axonal injury can be diagnosed histologically using immunohistochemical labeling for amyloid precursor protein (APP), which accumulates rapidly in axonal swellings and bulbs post-injury, likely indicative of impaired axonal transport (Suehiro and Povlishock, 2001; Smith et al., 2003). As diagnosis of DAI in humans can only currently be made through post-mortem investigations, there is growing interest in correlating diffusion tensor imaging with clinical presentations as a novel approach to identifying severity of axonal damage (Bazarian et al., 2007), however this area remains controversial (Ilvesmäki et al., 2014).

Although “diffuse” implies that it is widespread throughout the central nervous system (CNS), DAI is better described as lesions in multiple, yet common, loci throughout the white matter tracts (Smith and Meaney, 2000; Meythaler et al., 2001; Maas et al., 2008; Johnson et al., 2013), specifically the corpus callosum and within the cerebral hemispheres and brainstem (Adams et al., 1989). Rotational injuries in swine (Smith et al., 2000) and TBI in humans (Skandsen et al., 2010; Matsukawa et al., 2011) have demonstrated that axonal lesions within the brainstem and genu of corpus callosum are negative prognostic indicators, and that the location of DAI is related to severity and the plane in which the force is applied (Smith et al., 2000). Primary axotomy is a rare event; rather, cytoskeletal abnormalities that proceed to secondary axotomy are the most common form of DAI (Wolf et al., 2001; Stone et al., 2004; Chung et al., 2005; Kelley et al., 2006; Wang et al., 2011; Greer et al., 2013).

The human brain is viscoelastic in nature (Meythaler et al., 2001; McKee et al., 2014), and is mechanically compliant under normal, gradual accelerations associated with daily living. Due to inertia, sudden acceleration over an interval of less than 50 ms will overcome the brain’s viscoelastic properties, resulting in frank shearing of the cell membrane and cytoskeletal elements, often followed by a delayed elastic return to pre-injury morphology (Smith et al., 1999; Smith and Meaney, 2000; Tang-Schomer et al., 2010). The overall extent of DAI is amplified if the application of force persists once the elastic threshold of axons is surpassed (Meythaler et al., 2001). Although it is known that the axonal cytoskeletal network is subjected to shearing and torsional forces after a TBI, questions remain as to how this process triggers subsequent chemical and molecular changes. Although DAI is a frequent pathology seen in TBI, it is heterogeneous in nature, as not all axons that are subjected to the same forces display appreciable transport deficits (Johnson et al., 2013).

## Calcium mediates an injury cascade in diffuse axonal injury

**Figure 1:**
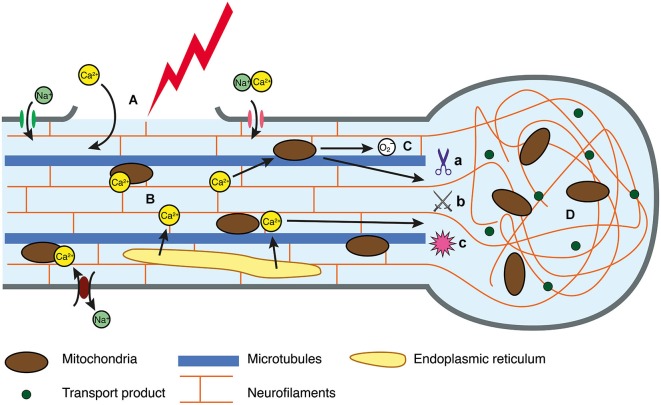
**Intracellular injury cascade in DAI. (A)** In response to trauma, the axolemma either undergoes primary mechanical failure, exposing the cytosol to the extracellular space, or mechanosensitive sodium channels are activated, resulting in a flux of sodium into the axoplasm. **(B)** Perturbation to the ionic equilibrium results in directional change in flow of calcium, resulting in intracellular accumulation. **(C)** Calcium can be sequestered in the mitochondria, however this generates reactive oxygen species that may disrupt oxidative metabolism and have downstream consequences with respect to oxidative damage to an axon in crisis. Similarly, elevated calcium can activate calcium-dependent calpains **(a)**, caspases **(b)** and phosphatases **(c)** all of which mediate cytoskeletal breakdown. **(D)** Cytoskeletal breakdown results in impaired axonal transport, axonal swelling and neurofilament compaction.

The exact mechanisms that initiate secondary degeneration in DAI are yet to be completely characterized, although *in vivo* and *in vitro* experimental models provide some insight. For example, fluid percussion models of injury in mice have reinforced the notion that mechanical stretching and disruption of the axolemma are a primary event, with axonal damage detectable at 2 h (He et al., 2004) and 4 h post-injury (Spain et al., 2010). *In vitro*, such damage precedes ionic imbalance (Smith et al., 1999), which may precipitate axonal swellings, secondary axotomy and Wallerian degeneration (Johnson et al., 2013). Axonal alterations may be driven by increases in intra-axonal calcium levels. In DAI, mechanical disruption creating breaches in the axolemma has been suggested as a mechanism of extracellular calcium entry (Farkas et al., 2006; Kilinc et al., 2009). However, activation of transmembrane calcium channels that mediate the extracellular influx may also be essential. Stimulation of mechanosensitive sodium channels by axonal deformation may reverse sodium/calcium transporters and activate voltage-gated calcium channels, culminating in the influx of extracellular calcium (Figure 1; Wolf et al., 2001). Other calcium channels that have been implicated include voltage-gated L-type and T-type calcium channels (Knoferle et al., 2010). However, there is also evidence for intracellular calcium release in axonal injury (Staal et al., 2010; Stirling et al., 2014). A study of long-term primary neuron cultures subjected to an axonal stretch injury observed a biphasic calcium elevation, and indicated that both intracellular and extracellular calcium contribute to the overall increase in axoplasmic calcium (Staal et al., 2010). The link between the release of extracellular and intracellular calcium stores will be an important focus of future research, with a recent study showing that expression of stromal interaction molecules may perpetuate elevated cytosolic calcium, as its suppression has been shown to improve survival after an axonal cut injury (Hou et al., 2014).

Several studies have investigated the effects of calcium on primary axotomy, demonstrating that calcium entry is essential for plugging the breached axon membrane and reconfiguring the cytoskeletal environment to a growth cone morphology, allowing for potential regeneration (Gitler and Spira, 1998; Kamber et al., 2009; reviewed in Bradke et al., 2012). The effects of calcium alterations in DAI are less well defined. The source and level of calcium concentration can affect the cellular response to calcium alterations, particularly as calcium is known to have a biphasic reaction in many signaling pathways with activation at low concentrations and inhibition at high concentrations (Berridge et al., 2003). In axons that do not undergo primary axotomy, high levels of cytosolic calcium could damage axons or kill neurons through destruction of cytoskeletal elements (Staal et al., 2010; Liu et al., 2014) and mitochondrial dysfunction (Wolf et al., 2001), increasing the risk of progression to secondary axotomy. The different sources of calcium may also reflect a differential response to axonal injury. For example, the influx of calcium that occurs secondary to changes in axolemmal permeability may contribute to spectrin-mediated destruction in the subaxolemmal domain (Czeiter et al., 2009), whereas the release from intracellular stores may contribute more to the destruction of the core cytoskeleton. Increased calcium also facilitates excitatory neurotransmitter release to create a positive feedback loop, exacerbating calcium influx and silencing neurons through perpetual depolarization (Barkhoudarian et al., 2011). The resultant excitotoxicity may also trigger axonal degeneration, caspase activation (Hosie et al., 2012; King et al., 2013) and distal axon swelling (King et al., 2007).

In an attempt to correct the ionic imbalance, active transport is upregulated, which in turn increases glucose metabolism (Blennow et al., 2012). This places strain on the system, since mitochondria sequester calcium to counter the cytosolic excess (Buki et al., 2000), which generates reactive oxygen species and disrupts oxidative metabolism (Figure 1; Prins et al., 2013) through unknown mechanisms. The role of this stress in the injury cascade is also unknown (Maas et al., 2008; Peng and Jou, 2010), but given the role of mitochondria in the extrinsic apoptotic pathway (Xiong et al., 2014), it is likely that mitochondrial protection would lessen the progression of vulnerable axons on to secondary axotomy.

## Secondary injury cascade precedes cytoskeletal destruction

Mitochondrial dysfunction after calcium sequestration may represent one of the final stages of the injury cascade. This is evidenced by the colocalization of cytochrome *c* released by stressed mitochondria, with neurofilament accumulations and axonal swellings post-injury (Staal et al., 2007) where it may augment the apoptotic cascade (Bossy-Wetzel and Green, 1999; Buki et al., 2000). Administration of cyclosporin-A, a protective agent limiting cytochrome *c* release from mitochondria (Suehiro and Povlishock, 2001), significantly reduces the development of axon pathology and progression of injured axons to secondary axotomy (Staal et al., 2007). Dysfunctional mitochondria may also have a role in activating caspase, which in tandem with calpain and phosphatase activation may alter cellular metabolism and cytoskeletal elements (Saatman et al., 2010; Ma et al., 2012; Smith et al., 2013; Liu et al., 2014). Cytoskeletal alterations such as neurofilament compaction and disruption, and disassembly of the microtubule array, are thought to cause defective axonal transport and swelling (Smith and Meaney, 2000), although these appear to be separate phenomena, as argued below. Indeed, varicose axonal regions are spanned by both intact and fractured microtubules, suggesting that even partial disruption of the microtubule network is sufficient to trigger swelling (Tang-Schomer et al., 2012) and contributes to an increased risk of secondary axotomy in injured axons (Smith and Meaney, 2000). Although the role of microtubules in cytoskeletal disruption has been extensively characterized, less is known concerning the role of neurofilament proteins. However to explore this, it is relevant to first consider the structure of neurofilament proteins and their post-translational modifications.

## Neurofilaments are a major cytoskeletal element in axons

Neurofilaments are the key intermediate filaments (IFs) in neurons and a major component of the axonal cytoskeleton (Perrot et al., 2008; Fletcher and Mullins, 2010). Neurofilaments are widely but not ubiquitously expressed in the central and peripheral nervous systems, labeling distinct subpopulations throughout the brain and spinal cord (Paxinos et al., 2009; Sengul et al., 2013) and restricted to a subset of neurons in the neocortex (Campbell and Morrison, 1989; Kirkcaldie et al., 2002; Paulussen et al., 2011). There are five classes of IFs, which provide fundamental cellular infrastructure (Petzold, 2005). These proteins are differentially expressed between cell types and express tissue specific functions. Neurofilaments, the type IV IFs, are integral to somatic, dendritic and axonal morphology and function. Structurally, NFs are obligate heteropolymers assembled from four identified fibrous subunits (Nixon and Yuan, 2011). The major neuronal IFs in the CNS are those assembled from the NF triplet proteins: neurofilament light (NFL; 61kDa), medium (NFM; 90kDa) and heavy (NFH; 115kDa). These subunits share a highly conserved central rod domain by which they polymerize (Lariviere and Julien, 2004; Nixon and Yuan, 2011). The head and C-terminal tail domains are divergent between subunits, with the tail domains demonstrating the greatest heterogeneity, particularly in the residue length and number of phosphorylation sites (Perrot et al., 2008; Chang et al., 2009). Neurofilament medium and NFH have a greater number of residues in their tail constructs, 504 and 607 respectively, compared to NFL with only 143 (Chang et al., 2009). One of the key differentiating features between subunits is the number of Lys-Ser-Pro (KSP) repeat motifs in the tail domain of NFM and NFH (Perrot et al., 2008). KSP repeats appear to be functionally significant, as they are the primary sites of phosphorylation (Jaffe et al., 1998; Veeranna et al., 1998; Perrot et al., 2008; Yuan et al., 2012).

While NFL can form a homopolymer *in vitro*, NFs are obligate heteropolymers *in vivo* and must include NFL (Lee et al., 1993; Carter, 1998; Elder et al., 1998; Hirokawa and Takeda, 1998). Another neuronal type IV IF—α-internexin, 66kDa—is expressed developmentally prior to the appearance of mature NFs (Kaplan et al., 1990; Fliegner et al., 1994). Although α-internexin is widely expressed in the adult CNS and can coassemble with the triplet proteins to form NFs (Yuan et al., 2006; Nixon and Yuan, 2011), human and rat studies suggest that it can be expressed by a distinct population of neurons with little immunoreactivity for the NF triplet (Dickson et al., 2005).

Neurofilaments are particularly long-lived proteins with NFL half-life estimated at approximately 3 weeks (Millecamps et al., 2007). They are predominantly synthesized in the soma, with axon-directed monomers traversing the axon at 0.2–1 mm/day (Lariviere and Julien, 2004) on average, although movement is thought to be highly asynchronous and bidirectional with periods of fast transport up to 50–100 mm/day interspersed with stationary periods (Nixon, 1998; Prahlad et al., 2000; Roy et al., 2000; Liu et al., 2004; Yuan et al., 2009). This slow transport contributes to the long-term stability and integrity of the axonal NF network (Liu et al., 2004). The rate of axonal NF transport is dependent on the cytoskeletal environment, and transport is faster in axons with low NF content (Millecamps et al., 2007). At the axon terminal, NFs undergo calpain-mediated degradation, and calpain inhibition results in NF accumulations here (Roots, 1983). However, proteolytic breakdown of NFs can take place across the entire length of an axon (Millecamps et al., 2007).

The function of NFs is not fully understood. Although the importance of IFs for mechanical strength has been long recognized, the varied composition and regulation of neurofilaments suggest that they have a more complex role (reviewed in Capano et al., 2001). However, it is clear that their structural heterogeneity is likely to play an important role in dictating function.

## Posttranslational modifications of neurofilaments

Neurofilaments undergo a number of posttranslational modifications, including phosphorylation and glycosylation. Phosphorylation has been well characterized (reviewed in Dale and Garcia, 2012) and appears to be location dependent, with high levels of phosphorylation occurring in the internodal regions of axons and lower levels of phosphorylation in perikarya (Nixon et al., 1994; Liu et al., 2004). This is also reflected in the differential concentration of NFs throughout the neuron, where phosphorylation state regulates anterograde transport (Shea and Chan, 2008). NFL N-terminal phosphorylation, regulated by protein kinase A, takes place soon after synthesis and inhibits mature NF assembly within the soma (Nakamura et al., 2000; Zheng et al., 2003). In the axon, NFM and especially NFH become highly phosphorylated at multiple KSP sites on the tail domains, reconfiguring them into side-arms that extend from the assembled filament core (Veeranna et al., 1998; Dashiell et al., 2002; Stevenson et al., 2011). Studies in rats have demonstrated that neurofilament is phosphorylated by kinases directed to serine-proline residues specifically in the KSP repeat sections of both NFM and NFH, including cyclin-dependent kinases (cdk), members of the mitogen-activated protein kinase (MAPK) family, as well as glycogen synthase kinases (Jaffe et al., 1998; Veeranna et al., 1998). MAPK-mediated NF phosphorylation is one aspect of its diverse role in promoting neurite development and survival under physiological and stress conditions (reviewed in Neary, 2005; Roskoski, 2012). Of particular interest, activation of MAPK pathways has been observed in both *in vivo* and *in vitro* models of trauma, although the effects of this upregulation are not yet characterized (Dash et al., 2002; Mori et al., 2002; Hollis et al., 2009; Yu et al., 2010).

It is thought that phosphorylation at these sites, particularly in NFM (Jacomy et al., 1999; Rao et al., 2002) increases NF spacing due to negative charge repulsion (de Waegh et al., 1992). This is consistent with their high abundance in specific populations of axons, correlating with axonal caliber and therefore conduction velocity. NF sidearm phosphorylation promotes radial growth of the entire axon, and bridges neurofilaments to cytoskeletal elements, specifically actin and microtubules, as well as the axolemma (Elder et al., 1998; Hirokawa and Takeda, 1998; Chen et al., 2000; Chang et al., 2009; Nixon and Yuan, 2011). Phosphorylation of C-terminal domains also provides NFs with resistance to proteolysis (Schumacher et al., 1999; Huh et al., 2002; Lee et al., 2014), thus maintaining the integrity of the NF network across long axonal processes.

Neurofilament phosphorylation has also been intimately tied to myelination and axonal transport (de Waegh et al., 1992). In addition, investigations utilizing myelin associated glycoprotein (MAG)-null mice have shown significant reductions in the phosphorylation of NFM and NFH tails, which correlate with smaller axonal diameters (Yin et al., 1998). It is proposed that MAG interacts with an axonal receptor that activates cdk5 and ERK1/2, kinases that are both capable of phosphorylating NFM and NFH (Dashiell et al., 2002). This suggests that MAG-activated signaling may regulate NF phosphorylation, although this is yet to be fully elucidated. More recently, Monsma et al. (2014) have shown that myelinating cells can regulate the rate of NF transport, resulting in local accumulation and expansion of the axonal diameter (Monsma et al., 2014), a process for which phosphorylation is most likely the mediator, given its upregulation during the process of myelination and studies such as Lee et al. (2014), who demonstrated that MAPK cascades control anterograde transport of NFs. NF content does not determine the overall level of myelination: there are no significant differences in the expression of MAG between wildtype and NF knockouts (Wu et al., 2008). Although there appears to be no direct relationship between the level of myelination of an axon and its NF content, the level of myelination may nonetheless affect the level of axonal NF phosphorylation. This may explain why unmyelinated axons appear selectively vulnerable to secondary axotomy after *in vitro* stretch injury (Staal and Vickers, 2011), since NF phosphorylation appears to protect against enzymatic degradation (Huh et al., 2002).

Compared to phosphorylation, the role of NF glycosylation is less well studied. The head region of NFL and NFM are modified by the addition of O-linked N-acetylglucosamine moieties, while glycosylation occurs at the KSP repeats of NFH (Dong et al., 1993). These sites have a critical role in NF assembly and it is thought that glycosylation may have a role in the trafficking of NFs (Petzold, 2005).

## Neurofilament changes in injury

NF accumulation in injury may develop out of primary mechanical failure of the NF network (Meythaler et al., 2001). However, alterations to NFs also represent an early event in the development of DAI, preceding microtubule fracturing and depolymerization (Fournier et al., 2014). Impact acceleration and fluid percussion injuries have been demonstrated to result in reductions in the interfilament spacing post injury, either due to altered phosphorylation or proteolysis of the side arms (Povlishock et al., 1997; Okonkwo et al., 1998). These changes are commonly termed NF compaction. It is hypothesized that reductions in side-arm length reduce interfilament spacing and lead to NF network collapse. Side-arm loss also facilitates the access of RM014, an antibody which recognizes the NF rod domain, and is therefore widely used to identify NF compaction (e.g., Stone et al., 2001). Given that side-arm extension relies on C-terminal phosphorylation (Nixon et al., 1994; Chang et al., 2009), it follows that pathology influencing phosphorylation state may be a driver behind NF compaction.

Pathways that may mediate side-arm shortening or loss include calcium-dependent calpain and calcineurin activation, which may be consequences of calcium accumulation post-injury. Evidence supporting calcineurin-mediated dephosphorylation as a factor in NF compaction comes from trauma studies investigating the impact of known calcineurin inhibitors. *In vivo* and *in vitro* models of injury employing tacrolimus and cyclosporin A have demonstrated reductions in axonal pathology (Okonkwo and Povlishock, 1999; Marmarou and Povlishock, 2006; Staal et al., 2007; Dileonardi et al., 2012). However, it is important to consider that cyclosporin A has an established role in protecting mitochondria following elevated intracellular calcium, (Peng and Jou, 2010), thus, there is potential for these agents to exert neuroprotection through an unidentified mechanism.

Similarly, evidence supporting calpain proteolysis of NF side-arms comes from a weight drop study demonstrating that administration of a calpain inhibitor prior to injury attenuated axonal pathology (Buki et al., 2003). Although NFs have been identified as a calpain substrate (Ma, 2013), calpains may also act to degrade sub-axolemmal anchoring proteins, specifically spectrin (Saatman et al., 2003), thus the observed reductions in axonal injury may reflect preservation of NF scaffolding rather than prevention of direct NF degradation. However, it is interesting to note that NF side-arm phosphorylation confers protection against proteolytic action (Huh et al., 2002), thus the synergistic action of calcium-activated calpains and phosphatases may clip NF side arms, culminating in disintegration of the axonal IF network (Povlishock et al., 1997; Buki et al., 2003; Ma, 2013).

Rotational head injuries in pigs have demonstrated that NF compaction in axonal varicosities contain high levels of all NF subtypes, although NFL accumulates before NFM and NFH, suggesting a temporal sequence of cytoskeletal breakdown or differential subunit transport (Chen et al., 1999). Injury-mediated NF compaction is also observed in immature rats, which have a lower NFH/NFM stoichiometry, suggesting that the loss of NFM side-arms may be the main driver behind NF compaction (DiLeonardi et al., 2009). *In vitro* studies using a fluid pressure pulse to induce a mild axonal stretch injury in unmyelinated axons have shown that by 48 h post injury, 51% of injured axons showed elevated SMI312 immunoreactivity for phosphorylated NFs (Chung et al., 2005). At 72 h post-injury, complete axotomy was observed at the injury site in 70% of axons, supporting the proposal that neurofilament alterations precede axotomy in DAI. This observation suggests that changes in the phosphorylation state of NFs can take place after injury (Chung et al., 2005), however the implications of this remain unclear. Defining the complete mechanisms behind NF compaction is yet to be achieved, however further investigation of side-arm phosphorylation post-injury will be essential (Saatman et al., 2009).

NF compaction has long been thought to contribute to disrupted axonal transport in the setting of injury. Microtubules are essential for axonal growth and transport (Nixon and Yuan, 2011), with fracturing or depolymerization of the transport machinery thought to contribute to this transport block. *In vitro* axonal stretch injury with subsequent transmission electron microscopy has demonstrated that microtubule breakage occurs in abnormally convoluted axons post-injury (Tang-Schomer et al., 2012). Interestingly, these breakage points corresponded to the varicosities that develop 3 h after injury (Tang-Schomer et al., 2012), providing evidence that microtubule disruption precedes swelling development. Measurement of this phenomenon immunohistochemically has been achieved using antibodies to the APP (Blumbergs et al., 1994), as it is a fast anterograde axonal transport product that rapidly accumulates at sites of transport disruption (Saatman et al., 2003). Accumulation of APP and organelles at these sites leads to progressive swelling, culminating in axotomy (Buki and Povlishock, 2006).

Although both NF compaction and microtubule disruption were thought to contribute to axonal transport block after trauma, a growing body of evidence suggests that NF compaction and defective transport, as measured by APP, are separate pathophenotypes of DAI. Tacrolimus, a selective inhibitor of calcineurin-activated phosphatases (Liu et al., 1991), attenuates axonal damage and progression to secondary axotomy in a subset of axons (Marmarou and Povlishock, 2006; Staal et al., 2010), suggesting that cytoskeletal damage and progression to secondary axotomy are due to dephosphorylation and protease activation caused by axolemmal perturbation (Povlishock et al., 1997; Schumacher et al., 1999; Huh et al., 2002; Marmarou and Povlishock, 2006). However, in rats subjected to a cortical controlled impact injury, this therapeutic approach was only effective for APP-labeled axons. Axons experiencing NF compaction, shown by rod-domain RM014 labeling, were not protected (Marmarou and Povlishock, 2006). Thus it appears that phosphatase activation cannot completely explain cytoskeletal disruption in injured axons undergoing NF compaction. Furthermore, DiLeonardi et al. (2009) showed in the same model that APP and RM014 labeled the same anatomical distribution at six and 24 h after injury, but labeling was never directly colocalised, suggesting that NF compaction and transport failure are discrete forms of axonal injury.

Although NF compaction and impaired axonal transport are largely distinct (DiLeonardi et al., 2009), these pathological features occur in the same tracts after injury (Creed et al., 2011). More recently, a study of the spatial distribution of NF compaction and impaired axonal transport in the corpus callosum and pyramidal tracts after TBI demonstrated that impaired transport is the prevalent injury phenotype in white matter tracts at 24 h post-injury (Kallakuri et al., 2012). In considering this, it is clear that DAI is heterogeneous, with axons experiencing a differential response that could depend on intrinsic factors or variability in force distribution and loads.

## Translating changes in neurofilaments to a clinical setting

Given the role of NFs in TBI and a range of neurodegenerative conditions, these proteins may be useful plasma biomarkers for axonal injury and neuronal damage (Petzold, 2005). It has been proposed that frank axonal transection or secondary axotomy results in the release of NF proteins into the extracellular compartment and subsequently the cerebrospinal fluid (CSF), and eventually the bloodstream (Petzold, 2005). Since phosphorylation of NFH C-terminals is specific to axons, detection of this epitope (pNFH) has been identified as a possible blood biomarker for measuring the extent of axonal injury (Anderson et al., 2008); indeed, after human TBI it rises significantly in both CSF and serum (Siman et al., 2009). Early animal work detected a serum rise in pNFH 6 h after controlled cortical impact, peaking at 24–48 h before gradually decreasing to baseline; the rise was significantly higher in more severe injuries, and correlated with cortical loss (Anderson et al., 2008). This pattern has also been recorded following blast TBI (Gyorgy et al., 2011). As there is differential content of pNFH in the CNS, with significantly higher concentrations seen in the long white matter tracts of the spinal cord when compared to the cerebral cortex (Anderson et al., 2008), interpreting changes in its serum concentration may need to be made on a background of medical imaging.

In a population of pediatric TBI cases, patients whose initial computed tomography scan showed DAI had significantly higher serum levels of pNFH compared to those without DAI (Žurek et al., 2011). Furthermore, patients who went on to die by the 6-month follow up were retrospectively shown to have had significantly higher pNFH serum levels than survivors on days two, three and four after admission (Žurek et al., 2011). This was one of the first human studies to demonstrate that serum NFs may be prognostically useful in CNS trauma, albeit less so with respect to outcome. Similarly, measuring serum pNFH in spinal cord injury has been shown to correlate with complete and incomplete sensorimotor loss, however its sensitivity is lost at lower grades of injury (Hayakawa et al., 2012). Although these findings already have potential clinical significance, there is scope for continued research into correlating pNFH serum concentrations with clinical observations, histopathology and neurological outcome (Tisdall and Petzold, 2012; Yokobori et al., 2013). For the latter, serum elevation of S100B, glial fibrillary acidic protein and neuron-specific enolase have been shown to significantly correlate with neurological deficit, although they are not specific to axonal damage (Žurek and Fedora, 2012). More recently, pNFH has been shown to stratify lower grades of injury, with significant rises in pNFH seen up to 3 days after a mild TBI when compared to non-injured controls (Gatson et al., 2014). Although pNFH is showing great promise as both a sensitive and specific marker of axonal injury after TBI, consideration of other NF isoforms may further stratify injury severity. NFL holds a lot of potential, as it appears to accumulate more rapidly than the other isoforms after injury (Chen et al., 1999; Li et al., 2010). In Alzheimer’s disease, amyotrophic lateral sclerosis and Guillian Barré Syndrome, NFL serum levels are significantly different from healthy controls and patients without neural degeneration (Gaiottino et al., 2013). Interestingly, a recent case report involving a concussed boxer showed marked CSF elevation of NFL that did not normalize until more than 30 weeks post-concussion, emphasizing that a far longer time-course of injury and recovery is biologically detectable (Neselius et al., 2014). This is important to consider in the setting of competitive sports, as functional recovery often occurs within 1–12 weeks after the insult (Karr et al., 2014), carrying with it the all-clear to return to a potentially traumatic situation.

## Conflict of interest statement

The authors declare that the research was conducted in the absence of any commercial or financial relationships that could be construed as a potential conflict of interest.
